# Measles Outbreak in an Unvaccinated Family and a Possibly Associated International Traveler — Orange County, Florida, December 2012–January 2013

**Published:** 2014-09-12

**Authors:** Tania A. Slade, Benjamin G. Klekamp, Edhelene Rico, Alvaro Mejia-Echeverry

**Affiliations:** 1 Florida Department of Health in Orange County; 2 Florida Department of Health in Miami-Dade County

The Florida Department of Health in Orange County (DOH-Orange) was notified by a child care facility on January 11, 2013, that a parent had reported that an attendee and three siblings were ill with measles. All four siblings were unvaccinated for measles and had no travel history outside of Orange County during the periods when they likely had been exposed. A fifth, possibly associated case was later reported in a Brazilian citizen who had become ill while vacationing in Florida. The outbreak investigation that was conducted at multiple community settings in Orange County, including at an Orlando-area theme park, identified no additional cases. The genotype sequence was identical for cases 2–5, and visits to the same theme park suggested an unknown, common exposure and link between the cases. Sources of measles exposure can be difficult to identify for every measles case. Measles should be considered in the differential diagnosis of febrile rash illness, especially in unvaccinated persons. Reporting a confirmed or suspected case immediately to public health authorities is critical to limit the spread of measles.

Measles cases in the United States are evaluated by state and local health departments using standard case definitions and classifications (1). For this investigation, the CDC definition of exposure period as 7–21 days before rash onset and the American Academy of Pediatrics 2009 Red Book definition for infectious period as 2 days before symptom onset through 4 days after rash onset were used (1,2).

## Case 1

An Orange County unvaccinated resident aged 10 years developed a fever (maximum temperature 104.5°F [40.3°C]) on December 25, 2012. Rash onset was on December 28. Additional symptoms included cough, coryza, and conjunctivitis. The patient was evaluated at a local pediatric urgent care clinic on December 29 and given a presumptive diagnosis of viral rash of unknown etiology. Activities during the exposure period of patient 1 included a family trip to an Orlando-area theme park on December 15 and school attendance through December 21. No travel history or ill contacts were reported. During the patient’s infectious period, attendance at a church on December 24 and 25, a visit to a health care facility on December 27 (unrelated to illness), and the visit to the pediatric urgent care clinic on December 29 occurred. Patient 1 did not attend school during her infectious period because of winter break. No clinical specimens were collected from patient 1.

## Cases 2, 3, and 4

Patient 2 (aged 7 years), patient 3 (aged 13 years), and patient 4 (aged 4 years) are all siblings of patient 1, and their illnesses were secondary cases in this outbreak. All four children in the family were unvaccinated; the parents had claimed a religious exemption to vaccination. Prodromal fever (≥101°F [≥38.3°C]) onset occurred on January 6, 2013, for patients 2 and 3, with patient 4 having onset of prodromal fever the next day. Rash onset in patients 2, 3, and 4 occurred on January 10, 11, and 12, respectively. Additional symptoms reported in all three cases included diarrhea, cough, coryza, and conjunctivitis. After onset of illness in all four of their children, the parents independently suspected measles infection and reported this to the children’s child care facility and school. Patients 2 and 3 did not attend school during their infectious periods. Activities during the infectious period included participation on a sports team by patient 2 during January 4 and 5 and attendance at child care by patient 4 on January 7, the date of fever onset.

## Public Health Laboratory Analysis

Nasopharyngeal specimens were collected from patients 2, 3, and 4, a urine specimen from patient 3, and a blood specimen from patient 2 on January 11 by staff from DOH-Orange and tested at the Florida Department of Health (DOH), Bureau of Public Health Laboratories. On Monday, January 14, the laboratory reported that the blood specimen was positive for measles-specific immunoglobulin M and all nasopharyngeal and urine specimens were positive for measles virus RNA by reverse transcription polymerase chain reaction. Testing at CDC confirmed identical sequences of measles genotype D8 for all three cases.

## Possibly Associated Case 5

On January 25, 2013, the Florida Department of Health in Miami-Dade County (DOH-Miami-Dade) was notified by CDC of a report from Brazil of a confirmed measles case in a Brazilian citizen who had visited Florida during his exposure and infectious periods. Patient 5, aged 20 years, sought medical care for same-day onset of rash and a 4-day history of fever, oral lesions, and conjunctival hyperemia at a Miami urgent care facility on December 30, 2012. With a discharge diagnosis of acute pharyngitis, patient 5 returned to Brazil on December 31, where he tested serum-positive for measles immunoglobulin M and positive for measles virus RNA from nasopharyngeal and urine specimens; genotype D8 was detected. Public health officials in Brazil reported to CDC that this patient had no documented history of measles vaccination and had also visited Orlando-area theme parks in Orange County during the December 14–21 timeframe; the theme parks were not identified. The genomic sequence from the patient in Brazil was identical to the sequences obtained from patients 2–4 in the United States, suggesting an epidemiologic linkage between these cases (Figure). A total of four secondary cases linked to patient 5 were identified in Brazil, and all had genotype D8 detected.

## Public Health Response

On January 11, 2013, for patients 2–4, guidance was provided to the parents to isolate the children at home during the remainder of their infectious periods. Before laboratory confirmation was received, actions were taken to ensure a rapid response to the outbreak, including briefing public information officers, querying the DOH electronic syndromic surveillance system for additional cases, and updating the health care provider alert letter, media press release, and measles exposure notification letters to contacts of the patients.

On January 14, after laboratory confirmation, a health alert was distributed to health care providers, including hospital and urgent care facilities in Orange County, Florida. In addition, contact investigations were initiated at identified community settings where there was a potential for disease transmission. Measles exposure notification letters were distributed to contacts in the identified settings, recommending isolation of persons with onset of symptoms clinically compatible with measles (with immediate notification of DOH-Orange), review of personnel vaccination status, and full vaccination. Additionally, the pediatric urgent care clinic and the urgent care clinic in Miami were encouraged to review its diagnostic and infection control policies for patients with rash and febrile illness. Because the exposure source for patient 1 was unknown, contact investigation was also initiated at the public school attended during the patient’s exposure period. School health logs were reviewed for all school days in December 2012 for compatible infection; however, none was identified. No exclusions were applied at any public school because patients 1–3 did not attend while infectious and no clinically compatible cases were identified during the investigation. Three unvaccinated child care contacts of patient 4 were excluded from child care attendance and voluntarily isolated through January 28, 2013. In total, measles exposure notification letters were sent to 528 students, 50 school faculty and staff, 24 sports team contacts, 15 pediatric urgent care contacts, six health care facility contacts, and 67 child care contacts. The notification letters were also distributed to an unknown number of church parishioners during the January 19 weekend services. No additional cases were identified.

Upon notification of a potential theme park link, contact was initiated with the Orlando-area theme park where the suspected exposure of patient 1, and possibly patient 5, occurred to ascertain if there were any theme park guest complaints or reports of rash illness from the beginning of December 2012 through January 2013. No rash illness had been reported among theme park guests or staff.

DOH-Miami-Dade followed up on potential disease transmission from patient 5 at the urgent care facility in Miami. The facility identified 14 patients and three staff members who were potentially exposed to the measles virus. All identified persons reported evidence of immunity to measles and had no compatible illnesses.

### Discussion

Measles is an acute, highly infectious, viral disease spread via large respiratory droplets and aerosolized droplet nuclei. In the United States, interruption of year-round endemic transmission of measles was documented in 2000 (3). This occurred as a result of high population immunity, attained through vaccination, and rapid public health response to cases. However, infants aged <12 months and unvaccinated children and adults remain at risk for acquiring and transmitting measles. Despite a high number of potential exposures in school, child care, and health care settings, no transmission occurred, highlighting that high population immunity can limit measles transmission and maintain measles elimination in the United States.

Measles is currently endemic in much of the world, including Europe, where genotype D8 is circulating, which provides an ongoing source of imported cases to the United States (4–8). Despite a detailed investigation, no source of exposure was found for patients 1 and 5, who became febrile on December 25 and 26, respectively, and their presence at a theme park with domestic and international attendees was suggestive of a common exposure at this location. Large congregate settings might be an important potential source of measles exposure in the United States. This outbreak also highlights the importance of molecular epidemiology. The identical D8 sequences from patients 2–5 was suggestive of an epidemiologic linkage or common source of exposure, although two separate importations from areas where this lineage is circulating cannot be excluded as an explanation.

What is already known on this topic?

High population immunity to measles, which has been achieved through vaccination, combined with rapid public health responses to cases, have resulted in the elimination of endemic measles in the United States since 2000. Failure to vaccinate is one of the most frequent preventable causes of measles outbreaks among U.S. children. Unvaccinated persons continue to be at risk for measles infection.

What is added by this report?

Four children in a Florida family were diagnosed with measles in January 2013. None of the four were vaccinated against measles, and none had traveled outside of Orange County, Florida, during the periods when they likely had been exposed. A fifth case of measles was later reported in a Brazilian citizen who had become ill while vacationing in Florida at the same time as the first Florida patient. The genotype was determined for three of the siblings and the Brazilian case; all were identical. The investigation detected no additional cases and suggested that visits to the same theme park might have resulted in an unknown, common exposure.

What are the implications for public health practice?

Sources of measles exposure can be difficult to identify for measles cases. Destinations popular among domestic and international visitors might serve as important sources of exposure. Children should be vaccinated against measles routinely. Clinicians should be educated on the recognition and diagnosis of measles and should consider measles diagnoses in persons with no or unknown vaccination history and compatible symptoms. Rapid identification is critical to effective public health response.

The misdiagnosis of patients 1 and 5 is a reminder that many health care providers are no longer familiar with the clinical presentation of measles and they need to maintain a high index of suspicion when a clinically compatible febrile rash illness occurs in an unvaccinated person. High vaccination coverage in the entire population and rapid, robust public health response to cases, which includes physicians immediately reporting suspected cases to public health agencies, appropriate isolation and specimen collection for both viral detection and genotyping as well as serologic testing, and thorough contact investigations, are necessary elements in the effort to maintain measles elimination in the United States.

## Figures and Tables

**FIGURE:**
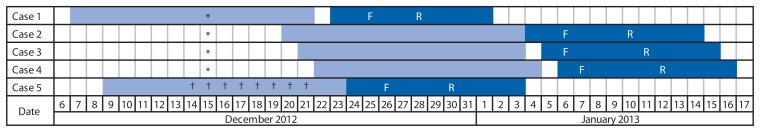
Exposure and infectious periods of five measles cases — Orange County, Florida, December 2012–January 2013 **Abbreviations:** F = fever onset; R = rash onset. * Known travel to a theme park in Orange County, Florida. ^†^ Known travel to Orange County, Florida, including visits to unknown theme parks. ^§^ Per CDC’s *Manual for the Surveillance of Vaccine-Preventable Diseases*. Atlanta, GA: US Department of Health and Human Services, CDC; 2013. Information available at http://www.cdc.gov/vaccines/pubs/surv-manual/chpt07-measles.html. ^¶^ Per American Academy of Pediatrics’ *Red Book* (Pickering LK, ed. Red book: 2009 report of the committee of infectious diseases. 28th edition. Elk Grove Village, IL: American Academy of Pediatrics; 2009:444–55). Information available at http://redbookarchive.aappublications.org/cgi/content/full/2009/1/3.77.
